# How Do You Think the Victims of Bullying Feel? A Study of Moral Emotions in Primary School

**DOI:** 10.3389/fpsyg.2019.01753

**Published:** 2019-07-30

**Authors:** Eva M. Romera, Rosario Ortega-Ruiz, Sacramento Rodríguez-Barbero, Daniel Falla

**Affiliations:** ^1^Department of Psychology, Universidad de Córdoba, Córdoba, Spain; ^2^High School Berenguela, Castilla La Mancha, Spain

**Keywords:** moral emotion attribution, victimization, cartoons, bullying types, gender

## Abstract

The important role of morality in the transgressive behavior which occurs within peer groups, such as bullying, has often been observed. However, little attention has been paid to this kind of violence in the initial stages of primary education. This study aims to analyze the attribution of moral emotions (self and other) to victims in different bullying types (verbal, physical, relational, and exclusion) and roles (aggressor and victim). An *ad hoc* questionnaire with supporting stick-figure cartoons was used. In total, 1150 schoolchildren between the ages of 6 and 11 years took part in the study (50.3% girls). The results showed that over 80% of schoolchildren had been involved in any type of aggressive behavior, and that there were significant differences by gender, year, and involvement in self- and other-attributed moral emotions. Aggressors showed less shame in general. In self-attribution situations, there was a greater indifference in aggressors. Victims had less shame and greater indifference in self-attributions for verbal and physical aggression. Girls recognized higher percentages of guilt in victims. The main moral emotion in the first stage was shame. This tendency changed to guilt as the children got older in both situations. Results support the need for the study of moral emotions development of victims and aggressors. How the experience of being involved in bullying biases the moral interpretation toward from the feelings of the victim is discussed.

## Introduction

From an early age, human beings are capable of attributing emotional and intentional states to others, both by reading facial expressions and by understanding the nature of the situation (Newman and Newman, 2010; Pozzoli et al., 2017). It has been noted that from the age of 4 years, children are able to recognize facial expressions of primary emotions (sadness, anger, joy, fear, surprise, or disgust), and can understand the events which precede and cause them (Lagattuta et al., 1997), and that these skills are basic steps in the development of psycho-social adjustment (Trentacosta and Fine, 2010). However, emotions such as guilt, shame, or pride, linked to a subjective interpretation made by the individual in complex social situations, are acquired at a later stage (Bosacki and Moore, 2004). Moral emotions entail a greater cognitive competence in order to interpret other people’s feelings, as do making moral judgments of situations based on the consequences arising from the protagonists’ actions and internalizing moral standards and shared social norms (Eisenberg, 2000; Malti et al., 2013b). Around 4–5 years old, children have no difficulty understanding the acts of victimization as morally wrong from a cognitive point of view (Nunner-Winkler and Sodian, 1988).

The cognitive-evolutionary tradition has held sway in studies of morality ever since the works of Kohlberg (1984), Piaget (1932), and Turiel (1983), in which the part played by emotions has been given less importance (Malti and Dys, 2015). Against this cognitive-evolutionary perspective, recent studies have stressed how emotions play a major role in moral action as they serve as a precedent for moral judgments and help to promote adherence to one’s own moral standards (Buon et al., 2016). Malti and Krettenauer (2013) carried out a meta-analysis in which they observed how moral emotions predicted high levels of prosocial and low levels of antisocial behavior. Moral emotions therefore play a regulatory role in social interaction, promoting or inhibiting maladaptive behavior and attitudes, as is the case of bullying in schools (Barón et al., 2018).

### Moral Emotions and Bullying Among Peers

Bullying is an interactive manifestation of aggression which can be categorized as antisocial behavior, since, regardless of the greater or lesser degree of harm one or more schoolchildren can cause to another (the victim), there is always a factor of unjustified, malicious, harmful, and intentional violence which makes it immoral (Ortega, 2010). This immoral behavior is linked to serious consequences in all participants, mainly in victims, who may suffer mental health and social adjustment problems (Romera et al., 2016; Garaigordobil et al., 2019; Krusell et al., 2019; Naveed et al., 2019).

A considerable body of research has highlighted the connection between moral emotions and bullying behavior [see Romera et al. (2019) for a review]. Social behavior is particularly regulated by the twin emotions of shame and guilt, although there are important conceptual differences between them (Stuewig et al., 2010). Shame involves a negative evaluation of the self when faced with social and moral standards, whereas guilt relates to specific behavior which does not comply with these standards (Tracy and Robins, 2006; Tangney et al., 2007). The feeling of guilt stresses the negative consequences of aggressive acts and reduces the likelihood that they will occur again in the future (Arsenio, 2014). Pride involves an emphasis on public recognition and social dominance (Krettenauer and Casey, 2015), while indifference involves the absence of negative emotions when faced with transgressive behavior (Gini et al., 2014; Carrera-Fernández et al., 2018). Both emotions, pride and indifference, were considered by some authors as self-evaluating emotions of moral disengagement arising from a transgression, which reveals the absence of empathy toward the victim through mechanisms of moral disengagement (Caurcel and Almeida, 2008). In the case of the aggressors in bullying, they showed greater degrees of pride and indifference (Menesini et al., 2015) and lower levels of shame and guilt (Mazzone et al., 2016).

Research has shown how girls tend to experience more guilt and shame than boys in situations of aggression (Walter and Burnaford, 2006; Roos et al., 2011). On the other hand, boys are reported to show more pride when they are aggressive toward others (Ferguson et al., 2000). As far as age is concerned, expressions of guilt seem to increase in frequency and intensity from early to middle childhood. This evolutionary trend has been linked to a gradual internalization of moral norms ranging from strict compliance to parental discipline or school norms through to adopting one’s own moral norms (Kochanska et al., 2002; Malti et al., 2013a; Herrera et al., 2016).

Most research into moral emotions and bullying has assessed how individuals feel after performing an immoral act toward their peers. However, it is also important to explore how school children understand the emotional repercussions of that aggression on their victims (Peplak et al., 2017). Studies of moral attributions stress the importance of differentiating between self-attribution (where schoolchildren are asked to put themselves in the victim’s position) and other-attribution (in which they are asked to assign an emotion to a victim other than themselves). In studies of emotional attribution to others, boys and girls more often refer to the victims using emotions of shame and guilt (Caurcel and Almeida, 2008; Gasser and Keller, 2009). As for aggressors, greater levels of indifference and moral disengagement toward the victims have been noted (Perren et al., 2012). These studies stress that schoolchildren tend to dehumanize and blame the victim, as a means of justifying and accounting for another people’s aggression (Garland et al., 2017; Thornberg and Wänström, 2018). In the research into self-attribution, the emotions of shame and guilt decrease and there is a marked rise in pride and indifference (Caurcel and Almeida, 2008). The different results found in these two types of attributions are due not so much to a deficit in cognitive abilities, but rather to a closer personal connection with self-attributed antisocial situations (Malti and Krettenauer, 2013). However, the majority of the studies were carried out in secondary schools (Caurcel and Almeida, 2008) or in the later years of primary school (Gasser and Keller, 2009) and up to now, very little attention has been paid to these attributional processes in younger boys and girls, at vital ages in the development and formation of moral criteria. It may be due to the fact that most of studies about moral attribution use self/hetero-report that requires comprehensive reading skills. The study of emotional moral attributions in younger children is very useful for the design of prevention programs adapted to moral emotions development in bullying, but requires the use of instruments adapted to them (Kutnick et al., 2007). Cartoons have been useful to measure aggressive behavior in young children (Huitsing and Monks, 2018). Likewise, although some recent studies have pointed out the moral and emotional differences in direct and indirect forms of bullying (Kokkinos and Kipritsi, 2018; Bjärehed et al., 2019), very few studies have focused on the possible differences in the emotional attribution to the victims and the different aggression types. There are good reasons for combining aggression types, because there is a strong conceptual overlap between the aggression types.

### The Aims of This Study

This study aims to analyze the attribution of moral emotions (to oneself and to others) in primary schoolchildren (6–11 years old) in the various manifestations of bullying processes among peers (verbal, physical, relational, and exclusion).

In particular, we set the following objectives:

(a) To analyze the differences in the attribution (self and other) of moral emotions in peer victimization depending on bullying type (verbal, physical, relational, or social exclusion) and role (aggressor or victim).
(b) To explore the differences in the attribution of moral emotions (self and other) to the victims of physical, verbal, relational aggression, and social exclusion in relation to gender and stage of schooling.

This study is based on the following hypotheses:

(a) Schoolchildren who admit to being victims of bullying will attribute more shame and guilt to the victims than those who are not involved.
(b) Girls will point out more moral attributions of guilt, while boys will express pride and indifference to the victims.
(c) Children in the early years of primary school will express a greater feeling of shame than the children in the later years.

## Materials and Methods

### Participants

A theoretical intentional sampling (Singleton and Straits, 2004) was used to select schools. Different characteristics of the context (type of center – public or private – and socio-economical level) were controlled to allow the adequate comparison of the data (principle of heterogeneity) (Valles, 1997). Eight schools were selected. All students of each school participated in the study. The incidental sample was made up of 1150 students (50.3% girls) in primary education, aged between 6 and 11 years old (*M* = 8.58, *SD* = 1.87), divided into in three educational stages (first 33.7%, second 31.4%, and third 34.9%).

### Instruments

We designed an *ad hoc* questionnaire in which different bullying incidents were narrated in a text illustrated with stick-figure cartoons, as used in previous research (Ortega-Ruiz et al., 2002; Monks and Smith, 2006; Huitsing and Monks, 2018). The first picture shows a situation of exclusion and had the following accompanying text: “Some children play football together every day. Another child asks if he/she can play, but every day the others say he/she can’t play with them.” The second illustrates physical violence and is accompanied by the following text: “A boy or girl goes to the playground in the break ready to play with their classmates, but then bumps into another boy or girl who kicks them for no apparent reason.” The third involves indirect relational violence, with its accompanying text: “A group of friends go out to the playground and criticize another child.” The fourth one focuses on verbal violence and has the following text: “A boy or girl goes out to the playground and starts to insult another boy or girl.” These stick-figure cartoons were taken from original works by Smith et al. (2002).

In order to understand the involvement of schoolchildren in bullying, we asked two questions which referred to the stick-figure cartoons: “Have you ever done it?” and “Have someone ever done it to you?.” The answers were “Yes/No.” Moral self-attribution was measured using the following question for each of the stick-figure cartoons: “Here are four emotions the boy or girl might feel when they are not allowed to play. How do you think they will feel?.” Four exclusive response options were given: *shame*, *guilt*, *pride*, and *indifference*. To measure moral self-attribution, the same response options were used for the question “How would you feel if you were that child?”

### Procedure

Once we had obtained permission for this research from the Ethic Committee for Bioethics and Biosafety at the University of Córdoba, a meeting was held with the school heads involved to inform them of aims of the study. We asked the children’s families for their consent in writing to participate in the study using a printed letter. The confidentiality, anonymity, and voluntary nature of the study were guaranteed throughout the process. All the interviews were supervised by the researchers involved in the study. The schoolchildren aged between 8 and 11 years answered the questions individually on paper during normal class time, while the 6- and 7-year-olds gave their answers orally, and this interview took place in a specially appointed room outside the classroom. Each child was shown the stick-figure cartoons referring to the different situations of bullying and the questions were read out loud. Their answers were written down by the interviewer.

### Statistical Analysis

Contingency tables with the chi-square statistic (χ^2^) were used. This non-parametric test was used according to the categorical variables of study. Adjusted standardized residual (ASR) values were taken into account > |1.96| y > |2.58| to check for significant differences. Cramer’s *V* index was included to note the strength of the association between the variables. SPSS software package version 20.0 was used to analyze the data. The level of significance was set at *p* < 0.05.

## Results

The data showed that out of the 1150 schoolchildren interviewed, 83.8% said that they had been involved occasionally in the behavior shown in the stick-figure cartoons, and most of them identified themselves either as victims or aggressors of verbal bullying (46.2% aggressors and 74.4% victims), followed by other forms of relational aggression (36% aggressors and 66.7% victims) (Table 1). 6.6% (*n* = 76) stated they had been involved in all the types of bullying as aggressors and 24.2% (*n* = 278) as victims.

**TABLE 1:** Percentage of roles of involvement and types of aggression manifested.

	**Aggressor**	**Non-aggressor**	**Victim**	**Non-victim**
E	28.2%	71.8%	64.5%	35.5%
	(*n* = 272)	(*n* = 692)	(*n* = 623)	(*n* = 341)
IR	36.0%	64.0%	66.7%	33.3%
	(*n* = 347)	(*n* = 617)	(*n* = 643)	(*n* = 321)
P	30.3%	69.7%	65.4%	34.6%
	(*n* = 292)	(*n* = 672)	(*n* = 630)	(*n* = 334)
V	46.2%	53.8%	74.4%	25.6%
	(*n* = 445)	(*n* = 519)	(*n* = 718)	(*n* = 246)

*E, exclusion; IR, indirect relational; P, physical; V, verbal.*

### Moral Attribution to Others and Involvement in Bullying

Significant differences were observed in other-attributed moral emotions between schoolchildren who admitted being involved as aggressors and those who did not, in each of the forms of bullying shown. Those who admitted to being aggressors showed less shame and more indifference toward the victims in the case of physical aggression, χ^2^(3.964) = 15,166, *p* = 0.002, and relational, χ^2^(3.964) = 12,343, *p* = 0.006. As regards of verbal bullying, lower levels of attribution of shame and higher levels of indifference were observed, as well as high levels of pride, χ^2^(3.964) = 34,344, *p* < 0.001. Cramer’s *V* values ranged between 0.11 and 0.18. No differences were observed in situations of exclusion (Table 2).

**TABLE 2:** Percentages of moral hetero-attribution and forms of bullying of aggressors.

	**Shame**		**Guilt**		**Indifference**		**Pride**		**Total**
	***n* (%)**	**ASR**	***n* (%)**	**ASR**	***n* (%)**	**ASR**	***n* (%)**	**ASR**	***n* (%)**
Exclusion (not involved)	299 (43.2%)	–	193 (27.9%)	–	183 (26.4%)	–	17 (2.5%)	–	692
Exclusion (involved)	93 (34.2%)		92 (33.8%)		77 (28.3%)		10 (3.7%)		272
Physical (not involved)	323 (48.1%)	3.0^∗∗^	239 (35.6%)	−0.3	93 (13.8%)	−3.5^∗∗^	17 (2.5%)	−0.2	672
Physical (involved)	110 (37.7%)	−3.0^∗∗^	107 (36.6%)	0.3	67 (22.9%)	3.5^∗∗^	8 (2.7%)	0.2	292
Indirect relational (not involved)	340 (55.1%)	3.4^∗∗^	144 (23.3%)	−1.4	118 (19.1%)	−2.2^*^	15 (2.4%)	−1.2	617
Indirect relational (involved)	151 (43.6%)	−3.4^∗∗^	95 (27.5%)	1.4	87 (25.1%)	2.2^*^	13 (3.8%)	1.2	346
Verbal (not involved)	289 (55.7%)	4.6^∗∗^	151 (29.1%)	−0.4	76 (14.6%)	−4.5^∗∗^	3 (0.6%)	−2.6^∗∗^	519
Verbal (involved)	181 (40.7%)	−4.6^∗∗^	135 (30.3%)	0.4	117 (26.3%)	4.5^∗∗^	12 (2.7%)	2.6^∗∗^	445

*^∗^Adjusted standardized residuals ≥1.96. ^∗∗^Adjusted standardized residuals ≥2.58.*

No significant differences were observed in the attributions to others by those involved in bullying.

### Moral Attribution to Others and Educational Stages

Higher percentages for shame were found in early primary age (age 6–8 years) in all kinds of situations of bullying (verbal, physical, relational, and exclusion), while these percentages decreased in subsequent years. In contrast, all the other moral emotions increased as the children got older. The differences were significant in all forms of bullying: exclusion χ^2^(6.964) = 73,107, *p* < 0.001; physical χ^2^(6.964) = 57,152, *p* < 0.001; relational χ^2^(6.963) = 18,652, *p* = 0.005; and verbal χ^2^(6.964) = 29,341, *p* < 0.001. Cramer’s *V* values were in the range 0.09–0.19 (Table 3).

**TABLE 3:** Percentages of moral attribution to others and the stage of schooling.

	**Shame**		**Guilt**		**Indifference**		**Pride**		**Total**
	***n* (%)**	**ASR**	***n* (%)**	**ASR**	***n* (%)**	**ASR**	***n* (%)**	**ASR**	***n* (%)**
Exclusion	154	6.6^∗∗^	37 (13.8%)	–6.7^∗∗^	76	0.6	1	–2.8^∗∗^	268
First stage	(57.5%)				(28.4%)		(0.4%)		
Exclusion	105	–3.8^∗∗^	110	2.1^*^	101	2.0^*^	9	0.0	325
Second stage	(32.3%)		(33.8%)		(31.1%)		(2.8%)		
Exclusion	133	−2.4^*^	138	4.1^∗∗^	83	−2.5^*^	17	2.7^∗∗^	371
Third stage	(35.8%)		(37.2%)		(22.4%)		(4.6%)		
Physical	169	7.0^∗∗^	55	–6.2^∗∗^	42	–0.5	2	−2.2^*^	268
First stage	(63.1%)		(20.5%)		(15.7%)		0.7%)		
Physical	126	–2.7^∗∗^	136	2.7^∗∗^	52	–0.4	11	1.1	325
Second stage	(38.8%)		(41.8%)		(16.0%)		(3.4%)		
Physical	138	–3.8^∗∗^	155	3.0^∗∗^	66	0.8	12	1.0	371
Third stage	(37.2%)		(41.8%)		(17.8%)		(3.2%)		
Indirect relational	162	3.6^∗∗^	44	–3.7^∗∗^	54	–0.5	8	0.1	268
First stage	(60.4%)		(16.4%)		(20.1%)		(3.0%)		
Indirect relational	158	–1.1	86	0.8	73	0.6	8	–0.6	325
Second stage	(48.6%)		(26.5%)		(22.5%)		(2.5%)		
Indirect relational	171	−2.3^*^	109	2.6^∗∗^	78	–0.1	12	0.5	371
Third stage	(46.2%)		(29.5%)		(21.1%)		(3.2%)		
Verbal	168	5.4^∗∗^	59	–3.2^∗∗^	38	–2.8^∗∗^	3	–0.7	268
First stage	(62.7%)		(22.0%)		(14.2%)		(1.1%)		
Verbal	145	–1.8	104	1.1	71	1.0	5	0.0	325
Second stage	(44.6%)		(32.0%)		(21.8%)		(1.5%)		
Verbal	157	–3.2^∗∗^	123	1.9	84	1.6	7	0.7	371
Third stage	(42.3%)		(33.2%)		(22.6%)		(1.9%)		

*^∗^Adjusted standardized residuals ≥1.96. ^∗∗^Adjusted standardized residuals ≥2.58.*

### Moral Attribution to Others in Bullying Among Peers and Gender

When analyzing the relationship between moral attribution to others and gender, the following results were seen: girls attributed more blame in exclusion χ^2^(3.964) = 13,526, *p* = 0.004; physical aggression χ^2^(3.964) = 9,441, *p* = 0.02; and relational aggression χ^2^(3.963) = 9,269, *p* = 0.02. Boys showed greater attributions of indifference in exclusion, χ^2^(3.964) = 13,526, *p* = 0.004, and of shame in physical aggression, χ^2^(3.964) = 9,441, *p* = 0.02. Cramer’s *V* values ranged from 0.09 to 0.11. No significant differences were found in the forms of verbal bullying (Table 4).

**TABLE 4:** Percentages of moral attribution to others and gender.

	**Shame**		**Guilt**		**Indifference**		**Pride**		**Total**
	***n* (%)**	**ASR**	***n* (%)**	**ASR**	***n* (%)**	**ASR**	***n* (%)**	**ASR**	***n* (%)**
Exclusion	216	1.1	126	–3.5^∗∗^	153	2.2^*^	16	0.7	511
Boys	(42.3%)		(24.7%)		(29.9%)		(3.1%)		(100%)
Exclusion	176	–1.1	159	3.5^∗∗^	107	−2.2^*^	11	–0.7	453
Girls	(38.9%)		(35.1%)		(23.6%)		(2.4%)		(100%)
Physical	248	2.4^*^	161	–3.0^∗∗^	89	0.7	13	–0.1	511
Boys	(48.5%)		(31.5%)		(17.4%)		(2.5%)		(100%)
Physical	185	−2.4^*^	185	3.0^∗∗^	71	–0.7	12	0.1	453
Girls	(40.8%)		(40.8%)		(15.7%)		(2.6%)		(100%)
Indirect relational	266	0.8	108	–2.8^∗∗^	118	1.5	18	1.2	511
Boys	(52.2%)		(21.2%)		(23.1%)		(3.5%)		(100%)
Indirect relational	225	–0.8	131	2.8^∗∗^	87	–1.5	10	–1.2	453
Girls	(49.7%)		(28.9%)		(19.2%)		(2.2%)		(100%)
Verbal	259	–	134	–	108	–	10	–	511
Boys	(50.7%)		(26.2%)		(21.1%)		(2.0%)		(100%)
Verbal	211	–	152	–	85	–	5	–	453
Girls	(46.6%)		(33.6%)		(18.8%)		(1.1%)		(100%)

*^∗^Adjusted standardized residuals ≥1.96. ^∗∗^Adjusted standardized residuals ≥2.58.*

### Moral Self-Attributions: Putting Themselves in the Position of the Victim

Statistically significant differences were observed between those who admitted to being aggressors and those who did not. Lower percentages of shame and greater indifference were found in exclusion χ^2^(3.964) = 8,518, *p* = 0.03; physical aggression χ^2^(3.964) = 25,359, *p* < 0.001; and relational χ^2^(3.964) = 16,664, *p* < 0.001. In verbal aggression, higher percentages of pride and indifference were found in those who admitted to being involved in bullying, but these were lower than attributions of shame, χ^2^(3.964) = 30,916, *p* < 0.001. Cramer’s *V* values ranged from 0.09 to 0.17 (Table 5).

**TABLE 5:** Percentages of moral self-attribution of aggressors and those not involved.

	**Shame**		**Guilt**		**Indifference**		**Pride**		**Total**
	***n* (%)**	**ASR**	***n* (%)**	**ASR**	***n* (%)**	**ASR**	***n* (%)**	**ASR**	***n* (%)**
Exclusion (not involved)	283 (40.9%)	2.2^*^	196 (28.3%)	0.5	202 (29.2%)	−2.5^*^	11 (1.6%)	–1.0	692 (100%)
Exclusion (involved)	90 (33.1%)	−2.2^*^	73 (26.8%)	–0.5	102 (37.5%)	2.5^*^	7 (2.6%)	1.0	272 (100%)
Physical (not involved)	312 (46.4%)	4.6^∗∗^	245 (36.5%)	–1.6	105 (15.6%)	–3.4^∗∗^	10 (1.5%)	–1.3	672 (100%)
Physical (involved)	89 (30.5%)	–4.6^∗∗^	122 (41.8%)	1.6	73 (25.0%)	3.4^∗∗^	8 (2.7%)	1.3	292 (100%)
Indirect relational (not involved)	336 (54.5%)	3.7^∗∗^	138 (22.4%)	–0.7	129 (20.9%)	–3.5^∗∗^	14 (2.3%)	–0.3	617 (100%)
Indirect relational (involved)	146 (42.1%)	–3.7^∗∗^	84 (24.2%)	0.7	108 (31.1%)	3.5^∗∗^	9 (2.6%)	0.3	347 (100%)
Verbal (not involved)	271 (52.2%)	3.7^∗∗^	140 (27.0%)	0.7	105 (20.2%)	–3.7^∗∗^	3 (0.6%)	–3.7^∗∗^	519 (100%)
Verbal (involved)	180 (40.4%)	–3.7^∗∗^	111 (24.9%)	–0.7	136 (30.6%)	3.7^∗∗^	18 (4.0%)	3.7^∗∗^	445 (100%)

*^∗^Adjusted standardized residuals ≥1.96. ^∗∗^Adjusted standardized residuals ≥2.58.*

In schoolchildren who had admitted to being victims of bullying, there were only significant differences in the self-attributions of physical aggression, χ^2^(3.964) = 10,059, *p* = 0.01; and verbal aggression, χ^2^(3.964) = 14,283, *p* = 0.003. In both forms of bullying, higher percentages of attribution of shame were observed in those who had never been victims and of indifference in those who had been victims, according to the ASR (Table 6). Cramer’s *V* values were between 0.01 and 0.12.

**TABLE 6:** Percentages of moral self-attribution of victims and those not involved.

	**Shame**		**Guilt**		**Indifference**		**Pride**		**Total**
	***n* (%)**	**ASR**	***n* (%)**	**ASR**	***n* (%)**	**ASR**	***n* (%)**	**ASR**	***n* (%)**
Exclusion (not involved)	128 (37.6%)	–	93 (27.4%)	–	117 (34.4%)	–	2 (0.6%)	–	340 (100%)
Exclusion (involved)	245 (39.3%)	–	176 (28.2%)	–	187 (30.0%)	–	16 (2.6%)	–	624 (100%)
Physical (not involved)	155 (46.4%)	2.2^*^	128 (38.3%)	0.1	48 (14.4%)	−2.4^*^	3 (0.9%)	–1.6	334 (100%)
Physical (involved)	246 (39.0%)	−2.2^*^	239 (37.9%)	–0.1	130 (20.6%)	2.4^*^	15 (2.4%)	1.6	630 (100%)
Indirect relational (not involved)	167 (52.0%)	–	79 (24.6%)	–	68 (21.2%)	–	7 (2.2%)	–	321 (100%)
Indirect relational (involved)	315 (49.0%)	–	143 (22.2%)	–	169 (26.3%)	–	16 (2.5%)	–	643 (100%)
Verbal (not involved)	136 (55.1%)	3.0^∗∗^	65 (26.3%)	0.1	41 (16.6%)	–3.5^∗∗^	5 (2.0%)	–0.2	247 (100%)
Verbal (involved)	315 (43.9%)	–3.0^∗∗^	186 (25.9%)	–0.1	200 (27.9%)	3.5^∗∗^	16 (2.2%)	0.2	717 (100%)

*^∗^Adjusted standardized residuals ≥1.96. ^∗∗^Adjusted standardized residuals ≥2.58.*

### Moral Self-Attribution and Cycle of Schooling

As regards the educational cycle, significant differences were found in all the manifestations of bullying studied: exclusion χ^2^(6.964) = 50,601, *p* < 0.001; physical χ^2^(6.964) = 37,116, *p* < 0.001; relational χ^2^(6.964) = 46,602, *p* < 0.001; and verbal χ^2^(6.964) = 37,686, *p* < 0.001. The ASR showed that shame was the most commonly identified emotion in the first stage of schooling for all forms of bullying. In contract, blame was the least commonly recognized emotion the first stage for all manifestations of bullying, although it was identified increasingly more in the higher cycles for the forms of exclusion and physical bullying. In verbal bullying, indifference and pride were commonly identified in the last educational stage (Table 7). Cramer’s *V* values ranged from 0.13 to 0.16.

**TABLE 7:** Percentages of moral self-attribution and the educative cycle.

	**Shame**		**Guilt**		**Indifference**		**Pride**		**Total**
	***n* (%)**	**ASR**	***n* (%)**	**ASR**	***n* (%)**	**ASR**	***n* (%)**	**ASR**	***n* (%)**
Exclusion	147	6.4^∗∗^	40	–5.6^∗∗^	78	–1.0	3	–1.1	268
First stage	(54.9%)		(14.9%)		(29.1%)		(1.1%)		(100%)
Exclusion	100	–3.6^∗∗^	112	3.2^∗∗^	107	0.7	6	0.0	325
Second stage	(30.8%)		(34.5%)		(32.9%)		(1.8%)		(100%)
Exclusion	126	−2.4^*^	117	2.0^*^	119	0.3	9	1.0	371
Third stage	(34.0%)		(31.5%)		(32.1%)		(2.4%)		(100%)
Physical	149	5.5^∗∗^	67	–5.2^∗∗^	49	–0.1	3	–1.1	268
First stage	(55.6%)		(25.0%)		(18.3%)		(1.1%)		(100%)
Physical	122	–1.8	136	1.7	62	0.3	5	–0.5	325
Second stage	(37.5%)		(41.8%)		(19.1%)		(1.5%)		(100%)
Physical	130	–3.3^∗∗^	164	3.1^∗∗^	67	–0.3	10	1.5	371
Third stage	(35.0%)		(44.2%)		(18.1%)		(2.7%)		(100%)
Indirect relational	180	6.6^∗∗^	40	–3.7^∗∗^	44	–3.7^∗∗^	4	–1.1	268
First stage	(67.2%)		(14.9%)		(16.4%)		(1.5%)		(100%)
Indirect relational	147	−2.1^*^	86	1.8	86	1.0	6	–0.8	325
Second stage	(45.2%)		(26.5%)		(26.5%)		(1.8%)		(100%)
Indirect relational	155	–4.0^∗∗^	96	1.7	107	2.4^*^	13	1.8	371
Third stage	(41.8%)		(25.9%)		(28.8%)		(3.5%)		(100%)
Verbal	159	4.8^∗∗^	57	−2.1^*^	52	−2.5^*^	0	–2.9^∗∗^	268
First stage	(59.3%)		(21.3%)		(19.4%)		(0.0%)		(100%)
Verbal	147	–0.7	93	1.3	80	–0.2	5	–1.0	325
Second stage	(45.2%)		(28.6%)		(24.6%)		(1.5%)		(100%)
Verbal	145	–3.8^∗∗^	101	0.7	109	2.5^*^	16	3.6^∗∗^	371
Third stage	(39.1%)		(27.2%)		(29.4%)		(4.3%)		(100%)

*^∗^Adjusted standardized residuals ≥1.96. ^∗∗^Adjusted standardized residuals ≥2.58.*

### Self-Attribution and Gender: Gender Differences in the Moral Attribution of Bullying

As regards gender, significant differences were found in the sample studied, as seen below: after seeing the sketch on exclusion, boys generally made attributions of indifference, while girls mainly attributed blame χ^2^(3.964) = 29,474, *p* < 0.001; for physical aggression, boys tended to mention pride χ^2^(3.964) = 9,017, *p* = 0.02, as they did in the case of the stick-figure cartoons showing relational aggression, χ^2^(3.964) = 11,729, *p* = 0.008. Cramer’s *V* values were between 0.09 and 0.17. No significant differences were found for the cartoon of verbal aggression (Table 8).

**TABLE 8:** Percentages of moral self-attribution and gender.

	**Shame**		**Guilt**		**Indifference**		**Pride**		**Total**
	***n* (%)**	**ASR**	***n* (%)**	**ASR**	***n* (%)**	**ASR**	***n* (%)**	**ASR**	***n* (%)**
Exclusion	205	1.0	107	–5.1^∗∗^	188	3.7^*^	11	0.7	511
Boys	(40.1%)		(20.9%)		(36.8%)		(2.2%)		(100%)
Exclusion	168	–1.0	162	5.1^∗∗^	116	−3.7^*^	7	–0.7	453
Girls	(37.1%)		(35.8%)		(25.6%)		(1.5%)		(100%)
Physical	207	–0.7	184	–1.4	106	1.9	14	2.1^*^	511
Boys	(40.5%)		(36.0%)		(20.7%)		(2.7%)		(100%)
Physical	194	0.7	183	1.4	72	–1.9	4	−2.1^*^	453
Girls	(42.8%)		(40.4%)		(15.9%)		(0.9%)		(100%)
Indirect relational	266	1.4	111	–1.0	115	–1.6	19	2.9^∗∗^	511
Boys	(52.1%)		(21.7%)		(22.5%)		(3.7%)		(100%)
Indirect relational	216	–1.4	111	1.0	122	1.6	4	–2.9^∗∗^	453
Girls	(47.7%)		(24.3%)		(26.9%)		(0.9%)		(100%)
Verbal	247	–	120	–	131	–	13	–	511
Boys	(48.3%)		(23.5%)		(25.6%)		(2.5%)		(100%)
Verbal	204	–	131	–	110	–	8	–	453
Girls	(45.0%)		(28.9%)		(24.3%)		(1.8%)		(100%)

*^∗^Adjusted standardized residuals ≥1.96. ^∗∗^Adjusted standardized residuals ≥2.58.*

## Discussion

After >30 years of research on bullying, today it is known that affective and moral life is deeply involved in this phenomenon. The interpretation that school children make of their own and other feelings reveals the moral conception that is built in the years of primary school. The ethical schemes move the social climate of the school and, in a world in crisis of solidarity and commitment to the needs of health and social welfare (Giorgi et al., 2015; Mucci et al., 2016), the construction of the moral criterion can be at risk. This work shows that bullying is a social phenomenon which is prevalent even in the early years of primary education, although it does not necessarily manifest itself in very serious cases (García et al., 2015). In general, the moral criterion is different when you have been a victim than when you have not. This implies a selfishness and a lack of moral sensitivity in school children that only makes them appreciate more ethically what happens to the victim when they have been previously victimized. What is clear is that most boys and girls at these ages recognize the phenomenon of bullying and assign moral emotions to the victims. This study has attempted to demonstrate that the moral attribution made by primary school children for the four commonest types of bullying (verbal, physical, relational, and social exclusion) depends to a large extent on the perspective from which they view and analyze the phenomenon. They adopt certain roles when they see the stick-figure cartoons representing these types of behavior, and this has a decisive influence on what they think the victim of bullying feels. We have analyzed the moral self-attributions and attributions to others made by primary school students for the victims of bullying in order to check whether the differences depend on the role they take (victim, aggressor, or not involved). Similarly, we have tried to describe the variations that seem to exist depending on the participants’ educational stage and gender.

The results show that most primary school students admit to having been involved, occasionally, in situations of verbal, physical, relational, and exclusion bullying. All of them recognize the situations, and 8 out of 10 acknowledge that they have been involved at some time in the behavior shown in the stick-figure cartoons. They tell us that verbal and relational aggression are the commonest forms of bullying in primary schools. These results are similar to those found in previous studies where self-report instruments were used (Zych et al., 2015; López-Castedo et al., 2018). The procedure used in this study let go deeper in the way of thinking and moral attribution of children aged 6–8 years, about whom there is little information available, mainly because they have only just learnt how to read or write. We may confirm from these results, however, that bullying in its simplest and most characteristic forms occurs frequently at these ages. In fact, most of them affirm to have previous experience of being victimized, and many have experience in using verbal, physical, relational aggression, or exclusion against one of their peers. Primary school children recognize that this behavior entails a moral transgression, but so far it has been difficult to explore these moral attitudes in detail, for many different reasons, one of them is the natural cognitive egocentricity existent at this age. The use of stick-figure cartoons, in which it is easy to externalize behavior where there is a clear transgressor, allows them to express their moral attributions and analyze the emotional shades of feeling they are able to recognize in the victim (Gutzwiller-Helfenfinger et al., 2010).

The commonest moral attributions made by primary school students about the emotions of the victims of bullying were shame and guilt, which is in line with previous work (Caurcel and Almeida, 2008). However, their recognition of these moral emotions is significantly affected when schoolchildren have had personal experiences in bullying. Children who admitted to having played the role of aggressor tended to produce less attributions of shame and more emotional attribution of indifference to the pain felt by the victim, except in the case of social exclusion. Gasser and Keller (2009), Menesini et al. (2003), and Perren et al. (2012) already recognized a greater moral disengagement in schoolchildren who admitted to being aggressors of their peers. The results presented therefore show a certain disengagement in the moral interpretation and recognition of the victim’s pain in cases of children with experience as an aggressor, even in cases where this experience was not either very prolonged or very serious. This may be because being aggressor is related with low levels of moral sensitivity. Perhaps the denial of guilt enables them to avoid emotional discomfort – or shame – when faced with these types of moral transgressions. On the other hand, students who admitted to having occasionally been victims of the kind of bullying situations shown in the stick-figure cartoons (verbal, physical, relational, and exclusion aggression) tended to make the same moral attributions as those who had never been through such an experience.

When schoolchildren were asked to put themselves in the victim’s position (self-attribution), there was a greater feeling of indifference among those who admitted to being experienced aggressors in bullying, even when it referred to occasions where they had not been involved. These differences were significant in the case of aggressors for all forms of violence. On the other hand, among those who admitted to having been bullied, the differences were significant in the attributions of verbal and physical aggression, but not in the relational ones. It seems, therefore, that experience of having been bullied is linked to the attribution of indifference, at least in the commonest and most direct forms of bullying. This differs with the results in attributions to others, where no emotional difference was observed between victims and non-victims of bullying. These results could imply that when it comes to putting oneself in the victim’s position, the viewpoint of those who have previously been victims is morally distorted, allowing them to distance themselves from the pain they might be suffering, at least in forms of direct violence (verbal and physical). Similar reflections were already mentioned in the studies by Caurcel and Almeida (2008) in which the use of cognitive distortions to justify the transgression is interpreted in terms of keeping up positive self-esteem, neutralizing guilt and avoiding cognitive and moral dissonance when faced with an act which harms others, and it allows the victims to minimize or deny their suffering. As regards the second objective, we looked at the emotional attributions of the three educational stages that make up primary education. The main moral emotion in the first stage was shame, although this tendency changed to guilt as the children got older. Both emotions reflect the recognition and assumption of sociomoral values and norms (Malti et al., 2013b), but guilt clearly requires more complex cognitive and emotional processes and was therefore more common in the later stages. This increase of guilt is linked also to a higher individual internationalization of own moral norms (Kochanska et al., 2002). Particularly, guilt is present in relational aggression, physical and social exclusion, and hardly appears at all in verbal aggression, which seems to stimulate very little moral attribution in schoolchildren. The frequent use of language riddled with insults and swear words may also blur their ethical qualification of this behavior. However, physical and relational aggression and social exclusion certainly do trigger a sense of guilt for the victim’s feelings, especially from 8 years old upward (Garland et al., 2017, Thornberg and Wänström, 2018). In a similar way, the older children assigned more pride, in cases of bullying, than the younger ones. The attribution of feelings of pride to acts of bullying obviously requires a moral disengagement which may result more from socialization and habituation to the phenomena of bullying. These results differ from those found by Malti et al. (2013a), who showed that there were no significant differences in the moral attributions about bullying according to age, although the children studied by Malti et al. (2013a) were of secondary school age (12–16 years). It may be that the understanding of the immoral component of social exclusion always stays with us once it has been acquired, which would account for the differences between primary and secondary schoolchildren. In the case of emotional self-attribution, the results show higher percentages of shame in the first educational stages and an increased sense of blame in the third stage, as well as in attributions to others. In self-attributions, however, there seems to be a greater moral disengagement from the relational and verbal forms of bullying and increased indifference among the older children.

As far as the differences between boys and girls are concerned, the attributions to others clearly show that girls recognize higher percentages of guilt in the victims for all kinds of bullying. Other studies (Menesini et al., 2003; Gini, 2008; Roos et al., 2011) have pointed out that girls attribute blame to the aggressors, but in this work, we have observed that they also blame the victims. This discovery is rather difficult to interpret, although other authors have understood it as an expression of stereotypes and gender biases (Walter and Burnaford, 2006; Else-Quest et al., 2012). In boys, exclusion is related with attributions of shame when bullying involves physical aggression, perhaps because males associate the humiliation suffered by the victim of physical bullying with shame. Previous studies highlight this relation as a result of the influence of male role stereotypes (Else-Quest et al., 2012).

As regards self-attribution, the results are similar to those found in attributions to others for exclusion: in other words, girls attribute more guilt and boys more indifference; as shown in other studies, boys showed more pride when they make a moral interpretation of physical and relational bullying (Menesini et al., 2003; Gini, 2008; Roos et al., 2011).

In short, in primary schoolchildren, having previous experiences as aggressor were linked to less attributions of shame and greater indifference in both and self- and other-attributions. This could be due to an attempt to justify the damage they are causing in their peers (moral disengagement). While the previous experience as a victim was not related to significant differences in the moral attributions in children. Likewise, this study is in line with other studies that show an increase in guilt and a decrease in shame with the age, while by gender girls show more emotions of guilt and boys of indifference and pride. This study highlights the risks of setting a moral criterion based on the lack of solidarity and sensitivity to the suffering of others of school children.

## Conclusion

This research has used the novel methodology of an interview and a questionnaire supported by stick-figure cartoons representing the four most frequent types of bullying (verbal, physical, relational, and social exclusion). It allowed us to analyze the attributions of moral emotions made by primary schoolchildren for the feelings experienced by a victim of bullying, from the age of 6 years, an age which up to now has been the object of very little research. It has been shown that primary school children interpret and evaluate aggressive bullying behavior as a moral transgression which triggers emotions such as guilt, shame, and indifference and even the pride of the aggressor. It is also clear that moral attributions of the phenomenon depend on one’s perspective, especially when the children have experience of being involved. Being an aggressor toward one’s peers, for instance, significantly biases the moral criterion toward the suppression of emotions such as shame, while being a victim leads to emotional indifference or disengagement from the harm they may be suffering, mainly in direct forms of bullying.

The limitations of this study should be taken into account in future research: firstly, we have not considered the frequency of violent behavior when defining victims and aggressors; in addition, the size of the effects of association between variables is rather low. It may be because bullying is a complex behavior and different variables are related with it. Other variables like peer support or antibullying programs in schools could be interesting to be considered in future studies to deep in this violent dynamic. However, this work represents important progress in understanding the moral impact of a morally unjustifiable phenomenon, as well as in how young schoolchildren understand it, showing that the experience of having been involved in bullying biases this moral interpretation toward disengagement from the victim’s feelings. Future research should continue to explore our understanding of these emotional attributions through the use of methodologies which may allow to pinpoint more exactly the nature of the ethical inference made by schoolchildren of a social problem which affects them in their daily life at school. The study of moral emotions attributions in bystander could be of interest in future studies.

## Data Availability

The datasets generated for this study are available on request to the corresponding author.

## Ethics Statement

This research was approved by the Ethic Committee for Bioethics and Biosafety at the University of Córdoba. We asked the children’s families for their consent in writing to participate in the study using a printed letter. The confidentiality, anonymity, and voluntary nature of the study were guaranteed throughout the process.

## Author Contributions

All authors listed have made a substantial, direct and intellectual contribution to the work, and approved it for publication.

## Conflict of Interest Statement

The authors declare that the research was conducted in the absence of any commercial or financial relationships that could be construed as a potential conflict of interest.
